# Non-surgical management of urinary incontinence in children

**DOI:** 10.1100/tsw.2009.144

**Published:** 2009-11-18

**Authors:** Barry Duel

**Affiliations:** Pediatric Urology, Cedars-Sinai Medical Center, Los Angeles, CA, USA

**Keywords:** urinary incontinence, neurogenic bladder, voiding dysfunction, anticholinergic

## Abstract

Urinary incontinence and neurogenic bladder are common in children, and can be difficult to treat. This themed issue includes contributions by experts in the management of these disorders. Dr. John Kryger discusses the nonsurgical management of neurogenic bladder in children with spina bifida. Drs. Lori Dyer and Israel Franco summarize the literature and their experience with the use of botulinum toxin in neurogenic and non-neurogenic incontinence in children. Dr. Paul Austin summarizes the use of alpha-adrenergic blockers. These drugs are primarily used to treat bladder outlet obstruction due to prostatic hyperplasia, but show great promise in the treatment of dysfunctional voiding in children.

Every day, pediatric urologists are faced with children who suffer with incontinence. This takes many forms, from the child with simple enuresis, up to the most challenging surgical cases, such as bladder exstrophy. While incontinence is not life threatening, it can cause terrible emotional distress for the child and the family. Therefore, effective management of these children should be a priority for all of us, since the reward, in the form of a happy, sociable child, is so worthwhile.

Quite frankly, the surgical management of urinary incontinence has not changed much since the 1950s, when the current techniques of bladder augmentation were first described. In many cases, the need for surgical management results from the failure of medical management to halt progression of the underlying disorder. This issue of *TSW Urology* focuses on the nonsurgical management of both neurogenic and non-neurogenic incontinence, since it is in that area that the most interesting and innovative changes have been happening.

First, John Kryger[1] gives a concise, but complete, overview of the management of the child with neurogenic bladder. Although some of these children are born with severe irreversible bladder damage, others develop it over time. It is these patients who can most benefit from a better understanding of the underlying disease, with the goal of preventing “good” bladders from turning “bad”. In many cases, modern medical management, including early intermittent catheterization and anticholinergic medications, can avert the need for later bladder augmentation.

Next, Lori Dyer and Israel Franco[2] review the literature and their own experience with botulinum toxin for management of neurogenic and non-neurogenic incontinence in children. Although FDA-approved for this indication in adults, it remains an off-label use in children, with many pediatric urologists reluctant to use it. Their work demonstrates that botulinum toxin is, in fact, very safe and effective when used properly, and may be able to avert or delay the need for aggressive surgical management in children with neurogenic bladder. In addition, it has been quite effective in neurologically intact children with refractory voiding dysfunction.

Finally, Paul Austin[3] reviews the use of alpha-adrenergic antagonists in the management of urinary incontinence in children. This is another area where pediatric urologists have sometimes been fearful to tread, considering the use of these drugs to be the province of adult urologists who are treating elderly men with enlarged prostates. Once again, Dr. Austin's article shows us that although this is an off-label use of the medications, they are extremely safe and effective in the management of children with dysfunction voiding and sphincter nonrelaxation.

We hope this issue of *TSW Urology* adds some useful tools to your clinical practice. Although these children can be challenging to manage at times, it is always rewarding when a nonsurgical intervention is able to get them dry and happy.
